# Lyme Disease Presenting with Multiple Cranial Nerve Deficits: Report of a Case

**DOI:** 10.1155/2016/7218906

**Published:** 2016-08-21

**Authors:** Abhishek Chaturvedi, Keith Baker, Donald Jeanmonod, Rebecca Jeanmonod

**Affiliations:** St. Luke's University Hospital, 801 Ostrum Street, Bethlehem, PA 18015, USA

## Abstract

Lyme disease is a tick-transmitted multisystem inflammatory disease caused by the spirochete* Borrelia burgdorferi*. With more than 25,000 CDC reported cases annually, it has become the most common vector-borne disease in the United States. We report a case of 38-year-old man with Lyme disease presenting with simultaneous palsy of 3rd, 5th, 7th, 9th, and 10th cranial nerves.

## 1. Case

A 38-year-old man with a past medical history of hypertension and non-insulin-dependent diabetes presented to an Emergency Department (ED) in the Northeast United States in late summer complaining of left sided facial droop and numbness that he noted 5 hours ago upon waking up for the day. One month prior to presentation, the patient had had some flulike symptoms with low grade fevers, myalgias, arthralgias, and fatigue associated with a macular, blanching rash on his thigh. This rash was ovoid, about 12 cm in its greatest dimension, without central clearing. He developed a diffuse headache 1 week after his initial symptoms which was nonradiating but associated with intermittent blurry vision. The patient was seen by his primary care doctor at that time and had a negative test for Lyme disease. He was treated for cellulitis with cephalexin for 5 days. His rash, fever, and myalgias resolved after about 10 days, but he had continued to have the headache.

The patient's past medical history was notable for chronic neck and back pain, but he denied any new neck or back pain or stiffness. There was no history of recent head trauma, nausea, vomiting, seizures, difficulty swallowing/breathing/speaking, vision changes, weakness/tingling in the extremities, sick contacts, chest pain, and shortness of breath. He had no recent travel and did not recall any insect bites.

On examination, the patient had normal vital signs. His neurologic exam was remarkable for decreased sensation to fine touch and temperature on the left side of his face in the entire distribution of cranial nerve V. The patient had incomplete left sided facial weakness with forehead sparing with inability to close his left eye independently. He had mild rightward gaze palsy with left eye delay and incompleteness in tracking in all directions and decreased left sided palatal elevation with rightward uvula deviation. Deep tendon reflexes and the remainder of his exam were unremarkable.

The working differential diagnosis for the patient included multiple sclerosis, vascular insult, diabetic neuropathy, intracranial mass, dural venous sinus thrombosis, and Lyme disease. The patient's complete blood count and all chemistry studies were normal. He had a d-dimer of 1424 (reference range 0–424). The patient's CT showed no abnormalities, and the patient underwent urgent MRI and MRV of the brain with contrast which was also normal. Given his unremarkable workup, the patient was presumptively treated for Lyme with doxycycline pending his confirmatory immunoassays.

One day after his evaluation, the patient's enzyme immunoassay (EIA) for Lyme antibodies revealed very high levels of IgM (17.37; Ref. range: 0.00–0.79) and IgG (17.06; Ref. range: 0.00–0.79). This was followed by positive Western blot, with 7 positive bands for IgG and 3 positive one for IgM. The patient underwent outpatient lumbar puncture over a week after initiation of antibiotics, which was EIA negative for Lyme with negative Lyme PCR and was otherwise unremarkable. He completed his 3-week antibiotic course and had resolution of all his neurologic deficits as well as his headache.

## 2. Discussion

Lyme disease is a tick-transmitted multisystem inflammatory disease caused by the spirochete* Borrelia burgdorferi*. With more than 25,000 cases reported annually to the CDC, Lyme disease has become the most common vector-borne disease in the United States [1–3]. Ninety-six percent of Lyme disease cases in the United States have been reported in high endemic New England and mid-Atlantic states, as well as Minnesota and Wisconsin [1]. It is transmitted by the bite (salivary secretions) of infected Ixodes ticks, namely,* Ixodes scapularis* in the United States, and typically requires 24–48 hours of tick attachment, although cases of transmission with less than 24 hours of attachment have been reported and are supported by other animal models [4, 5]. Unfortunately, most adult patients do not recall the tick bite as what happened in our case, because the* Ixodes* ticks are very small and either go unrecognized when attached to the skin or fall off after feeding [6].

Lyme disease occurs in stages, with a wide spectrum of clinical signs and symptoms at each stage. Incubation varies from 3 to 32 days, after which a characteristic enlarging target-like rash, known as Erythema migrans (EM), may be evident and accompanied by flulike symptoms of fever, headache, malaise, and myalgia [7]. This is stage 1. EM is a hallmark of the disease and manifests as an area of expanding erythema >5 cm in diameter [2]. It may or may not have central clearing and can have vesicles or hemorrhage [8]. The finding of this rash on a patient in an endemic area is sufficient to make a diagnosis of Lyme disease and begin treatment, as EM is virtually 100% specific and more sensitive (57%–86%) than serology in a patient with early Lyme disease [9]. Migrating transitory musculoskeletal pain in limited joints, bursae, tendons, muscle, or bone is a common feature of early Lyme disease [10].

After several weeks to months, neurologic abnormalities and cardiac involvement may be seen in about 15% and 8% of patients, respectively [2]. This is stage 2. Lyme neuroborreliosis (LNB) can present as aseptic meningitis, recurrent meningoencephalitis, and cranial or spinal neuropathies, with the seventh cranial nerve being the most commonly involved [11–13]. There are few reports on cases of Lyme disease with more than one cranial neuropathy. Our case is unique, with 5 separate cranial nerves involved: third, fifth, seventh, ninth, and tenth. This illustrates an important feature of LNB: Lyme can involve any of the cranial nerves and can even be bilateral, which can cause diagnostic confusion, especially given the long lag time between tick bite and symptom occurrence [14]. This can make it easy to confuse with other central nervous system processes and delay diagnosis and appropriate treatment.

In stage 3, patients may develop chronic monoarticular or oligoarticular Lyme arthritis, which commonly involves large joints, particularly the knee [10]. It may also manifest as encephalomyelitis. Patients may have their initial presentation with Lyme disease occurring in any of the three stages.

Current standards for the diagnosis of Lyme disease include a sensitive EIA, followed by Western blot (or immunoblot) with findings of abnormal IgM and IgG antibodies. These tests are often negative if performed in the first two weeks of disease [7, 9]. IgM is only useful in early disease and may be absent after 6 weeks, while IgG typically becomes positive after 3–6 weeks. Several studies have shown that the intrathecal synthesis of Bb antibodies is of great importance for diagnosing LNB. The antibody index has a very high specificity (97%), but only a moderate sensitivity ranging from 40% to 89%. So the absence of specific immunoglobulins in the CSF does not exclude the LNB diagnosis [11, 15]. Attempt at isolating the spirochete by culture or PCR from blood or serum is unhelpful, as the spirochete rapidly redistributes to connective tissue and is not present in sufficient concentrations to be detected.

MRI may be helpful in the diagnosis of LNB or may be misleading. There are no published prospective studies citing the incidence of cranial or radicular nerve enhancement on MR imaging in the clinical setting of Lyme disease, but several reports have described high-signal lesions in the white matter on T2 weighted images, simulating demyelinating or ischemic disease [2]. MRI may be negative, as it was in our patient.

The treatment of Lyme disease varies depending upon the clinical picture. For uncomplicated Lyme disease a two-week course of doxycycline, 100 mg twice a day, is the first-line treatment for adults [16, 17]. Children over the age of eight can receive 4 mg/kg/day divided into twice daily dosing for two weeks [16, 17]. Doxycycline should not be given to patients who are pregnant or lactating, or to anyone under the age of eight. For these patients, as well as for those who are allergic to doxycycline, treatment with amoxicillin or cefuroxime is appropriate. Patients with Lyme arthritis can be treated with the same daily regimen but should be treated for four weeks instead of two [16, 17]. Symptomatic Lyme carditis requires hospitalization and treatment with intravenous antibiotics until symptoms have resolved, at which time transition can be made to oral antibiotics [16, 17]. Ceftriaxone, 2 g/day in adults and 50–75 mg/kg/day in children, is preferred for intravenous therapy, but cefotaxime can also be used. A temporary pacemaker may be necessary for patients who present with AV nodal dysfunction including third degree block.

As previously discussed, Lyme disease can present with a variety of neurologic manifestations. In these cases the decision to treat with oral versus parenteral antibiotics depends upon whether or not meningitis is present or suspected. In patients who have meningitis confirmed by CSF analysis from lumbar puncture, intravenous ceftriaxone is typically administered daily for two to four weeks in the US [16, 17]. Dosing is 2 g/day in adults and 50–75 mg/kg/day in children. In cases where Lyme meningitis is suspected but cannot be confirmed by CSF analysis, empiric treatment for meningitis is appropriate [16, 17]. There is some evidence that oral doxycycline may be as effective as parenteral antibiotics for the treatment of Lyme meningitis, but this evidence is from Europe and may not apply to other places where different species of* Borrelia* are present [13]. Still, given doxycycline's excellent CSF penetration, oral doxycycline does represent a treatment alternative for patients with Lyme meningitis who are either allergic to beta lactams or unwilling to undergo parenteral therapy.

In cases of Lyme disease where there are neurologic manifestations but meningitis is not suspected, treatment with oral antibiotics is appropriate [16, 17]. The treatment is the same as for uncomplicated Lyme disease but can be given for up to three weeks. It is important to educate patients that while antibiotics will likely treat the infection adequately, cranial nerve deficits may persist for weeks or months and can even be permanent. Corticosteroids can be used at the discretion of the treating provider, but there does not seem to be evidence which supports their efficacy in improving outcomes of cranial nerve deficits associated with Lyme disease.

## 3. Conclusion

Lyme disease may present in myriad ways and should be considered in the differential diagnoses of any isolated or multiple cranial neuropathy, especially in a Lyme endemic area. Emergency physicians and other acute care providers should be aware of the typical and atypical clinical symptoms of LNB. Early diagnosis and prompt establishment of adequate antibiotic treatment are important to prevent progression to further stages of disease.

## References

[1] http://www.cdc.gov/lyme/stats/.

[2] Hildenbrand P., Craven D. E., Jones R., Nemeskal P. (2009). Lyme neuroborreliosis: manifestations of a rapidly emerging zoonosis. *American Journal of Neuroradiology*.

[3] Centers for Disease Control and Prevention (CDC) (2007). Lyme disease: United States, 2003–2005. *Morbidity and Mortality Weekly Report*.

[4] Cook M. J. (2014). Lyme borreliosis: a review of data on transmission time after tick attachment. *International Journal of General Medicine*.

[5] Hynote E. D., Mervine P. C., Stricker R. B. (2012). Clinical evidence for rapid transmission of Lyme disease following a tickbite. *Diagnostic Microbiology & Infectious Disease*.

[6] Pachner A. R., Steiner I. (2007). Lyme neuroborreliosis: infection, immunity, and inflammation. *The Lancet Neurology*.

[7] Hengge U. R., Tannapfel A., Tyring S. K., Erbel R., Arendt G., Ruzicka T. (2003). Lyme borreliosis. *The Lancet Infectious Diseases*.

[8] Paul S., Song P. I., Ogbechie O. A. (2016). Vesiculobullous and hemorrhagic erythema migrans: uncommon variants of a common disease. *International Journal of Dermatology*.

[9] Tugwell P., Dennis D. T., Weinstein A. (1997). Laboratory evaluation in the diagnosis of Lyme disease. *Annals of Internal Medicine*.

[10] Steere A. C. (1995). Musculoskeletal manifestations of Lyme disease. *The American Journal of Medicine*.

[11] Radzišauskiene D., Ambrozaitis A., Marciuškiene E. (2013). Delayed diagnosis of lyme neuroborreliosis presenting with abducens neuropathy without intrathecal synthesis of borrelia antibodies. *Medicina*.

[12] Halperin J. J. (2010). Nervous system lyme disease. *Journal of the Royal College of Physicians of Edinburgh*.

[13] Mygland Å., Ljøstad U., Fingerle V., Rupprecht T., Schmutzhard E., Steiner I. (2010). EFNS guidelines on the diagnosis and management of European lyme neuroborreliosis. *European Journal of Neurology*.

[14] Vanzieleghem B., Lemmerling M., Carton D. (1998). Lyme disease in a child presenting with bilateral facial nerve palsy: MRI findings and review of the literature. *Neuroradiology*.

[15] Blanc F., Jaulhac B., Fleury M. (2007). Relevance of the antibody index to diagnose Lyme neuroborreliosis among seropositive patients. *Neurology*.

[16] Wright W. F., Riedel D. J., Talwani R., Gilliam B. L. (2012). Diagnosis and management of Lyme disease. *American Family Physician*.

[17] Shapiro E. D. (2014). Lyme disease. *The New England Journal of Medicine*.

